# Predictive Diagnosis Based on Predictor Symptoms for Isolated Photovoltaic Systems Using MPPT Charge Regulators

**DOI:** 10.3390/s22207819

**Published:** 2022-10-14

**Authors:** Emilio García, Eduardo Quiles, Antonio Correcher, Francisco Morant

**Affiliations:** Instituto de Automática e Informática Industrial, Universitat Politècnica de València, Camino de Vera, s/n, 46022 Valencia, Spain

**Keywords:** solar panel, predictive maintenance, fault diagnosis, photocell, partial shading degradation, hotspots

## Abstract

In this work, new results are presented on the implementation of predictive diagnosis techniques on isolated photovoltaic (PV) systems and installations. The novelties introduced in this research focus on the additional advantages obtained from the point of view of predictive diagnosis of faults caused by partial shading in isolated PV installations using maximum power point tracking (MPPT) regulators. MPPT regulators are comparatively more appropriate than pulse width modulation (PWM) solar regulators in order to implement fault diagnosis systems. MPPT regulators have a physical separation between the electrical parameters belonging to the part of the solar panel with respect to the batteries part. Therefore, these electrical parameters can be used to obtain early predictive symptoms of the effects of partial shading with a greater level of observation and sensitivity. Additionally, modifications are proposed in the PV system assembly to obtain greater homogeneity of all the panels regarding the solar irradiance reception angle.

## 1. Introduction

The appearance of hotspots in a photovoltaic installation (PVI) is a problem of great importance because it affects not only the production, but also the useful life of installation. The occurrence of hotspots produces premature degradation and aging of the photovoltaic modules (PVMs). This fact is worrying if one considers that PVMs are normally designed to function properly for more than 20 years. Photovoltaic hotspots, a well-known phenomenon for more than 50 years [1], still persist today [2,3]. Frequently, the appearance of hotspots occurs when a panel is partially exposed to shaded areas, and consequently, the affected cell or group of cells operates in reverse polarization conditions, dissipating energy instead of generating it. This condition favors the appearance of hotspots at very high temperatures, gradually degrading both the generated power and the components of the encapsulation material of the photovoltaic module [4].

One of the reasons for the greater frequency of the appearance of hotspots has been the tendency of PVM manufacturers to use wafers that are thinner but less resistant to the appearance of microcracks, with a greater propensity to develop them in the manufacturing, transportation, and even installation phases [5,6].

Reports from the UK have highlighted annual power losses of 18.9% associated with shadowing and persistent inverter failures [7]. In addition, in the “1000 rooftop PV systems” program implemented in Germany, it was recorded that the operation of the 41% of the installed PV systems had been affected by shading, with energy losses of the order of 10% [8]. The same results were found in a Japanese field test program [9]. In Spain, an investigation into hotspots in two large grid-connected PV plants was presented in [10].

In [11], a specific method based on the online analysis of the time-series data of random and seasonal I–V parameters was proposed for the comparative trend analyses of solar power generation focused on isolated installations using PWM charge regulators

The main purpose of a PV charge regulator is to ensure efficient and safe charging and discharging of batteries in order to prolong their useful life. PWM-type solar regulators help regulate the voltage coming from the photovoltaic panels to protect the system’s batteries from possible overcharges. When a solar installation has a PWM regulator connected, the regulator controls the battery charge by constantly checking the current state of the battery to adjust the delivery of the correct amount of charge.

**Figure 1:**
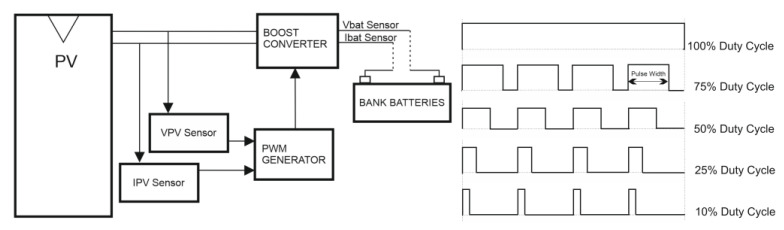
PWM solar charge regulators.

For this purpose, a PWM solar regulator emits on/off pulses to control the energy transfer. It also checks the battery status to determine the duration of the pulses and their frequency. That is, the PWM regulator essentially operates as a fast on/off switch. When the battery is nearly discharged, the pulses will be wide and continuous, while when the load increases, the pulses will be proportionally reduced or filtered (Figure 1).

PWM and MPPT regulators are two different types of solar charge controllers used to manage the electrical current between the battery, the solar panel, and the load. Both are commonly used in installations isolated from the general electrical network. However, the choice between a PWM regulator and an MPPT regulator depends on a diverse set of additional considerations such as the type of panels that are going to be used. If 60-cell panels are used or if the voltage of the photovoltaic field is higher than the voltage of the battery bank, an MPPT regulator is necessary. For small installations that deal with the lighting of a small house and that supply power to a few appliances, the use of a PWM regulator can be a cheaper solution. However, if the photovoltaic installation must provide a lot of power, the best option is to use an MPPT controller.

**Figure 2:**
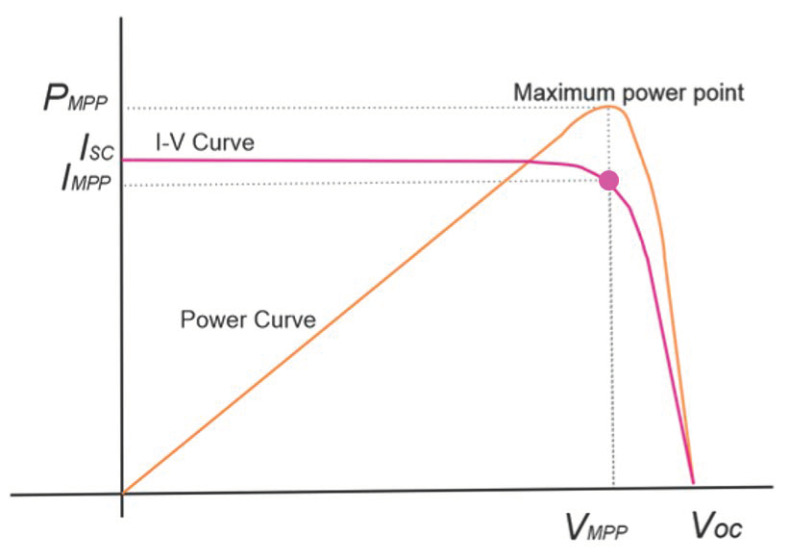
Maximum power point curve.

MPPT solar regulators reduce losses because they always work at the most appropriate voltage and are able to extract the maximum power and obtain a greater performance from photovoltaic modules (Figure 2). Additionally, they allow the use of solar panels that cannot be used with PWM regulators due to issues of compatibility between the voltage of the panels and the batteries. MPPT solar regulators also allow the addition of panels in series with a total voltage higher than that of the battery bank, which avoids the typical losses due to low voltage and high DC current.

The importance of the intersection point of the I-V values comes from the fact that the power reached is lower when the voltage is too high or too low. In other words, the point of maximum power is reached when the area of the lower square of the curves is the maximum.

In an initial analysis, MPPT regulators may show some disadvantages due to their higher initial cost, but if analyzed from a broader point of view, they have a series of additional characteristics that make them more convenient for certain applications. On the one hand, they usually house more complex and intelligent algorithms conducive to prolonging the useful life of the batteries. MPPT regulators are also more convenient when it comes to implementing predictive diagnosis techniques using the parametric analysis of the electrical power generated in the solar panel part.

**Figure 3:**
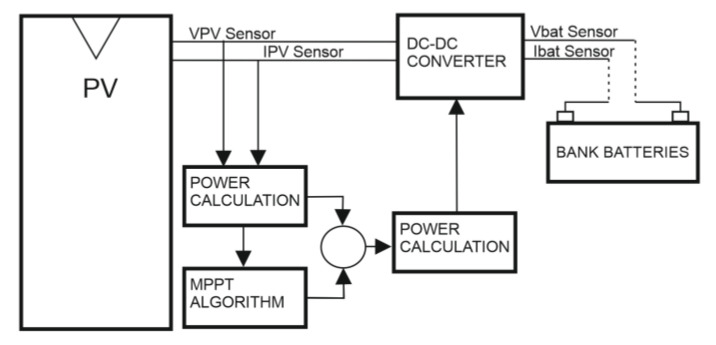
MTTP charge regulator block diagram.

In addition to a protection diode, an MPPT regulator, also called a solar maximizer, has a DC–DC voltage converter and a maximum power point tracker (Figure 3). This allows it to perform the following functions:

- The DC–DC voltage converter (from high voltage in the photovoltaic field to low voltage in the batteries) allows operation at different voltages in the photovoltaic field and in the batteries.
- The maximum power point tracker (MPPT) adapts the operating voltage in the photovoltaic field to the one that provides the maximum power.

Therefore, as in PWM regulators, in an MPPT regulator the energy that enters and leaves the regulator is the same but the voltage and current are different on each side, which increases the voltage of the solar panel and increases solar production by more than 30% compared to PWM charge regulators.

By being able to operate at higher voltages in the photovoltaic field, the energy losses caused by low voltages are reduced (since the losses are proportional to the current—the lower the current, the lower the losses), making MPPT regulators especially suitable for high photovoltaic power because they seek to generate the maximum energy.

To reduce possible losses that can occur in solar panels due to the phenomenon of partial shading, shade or power optimizers are used; these devices are part of the photovoltaic installation that help to maximize efficiency. The optimizer, unlike the inverter, has to be placed in each panel so that it can work individually and optimize the maximum power point separately. It does not transform the energy, but instead maximizes the direct current before redirecting it to the inverter [12,13].

The MPPT charge regulator is also known as a maximizer because its operation takes advantage of the maximum production of the solar panel, causing it to operate at its maximum point to charge the battery. It is the best option to obtain the best performance for PVMs and more than compensates for its possible extra cost. It can work with panels and batteries with the same nominal voltage but its operation is more efficient if the voltage of the photovoltaic field is increased [14,15].

In short, the advantages of using MPPT charge controllers are:

- Better performance than PWMs (10 to 40%).
- Better performance when there is partial shading.
- Better performance in case of low solar radiation.
- Any photovoltaic panel can be used.
- Lower voltage drops in installations in which the panels are far from the regulator when working with high DC voltage (250 V).
- Lower installation cost and lower wiring losses. By installing more panels in series, the number of parallels, the cable section, and the number of protections can be reduced.

On the other hand, if MPPT regulators are analyzed from the point of view of the possibilities of applying predictive diagnosis techniques, a very important issue to consider is the availability of parameters that MPPT regulators offer to carry out functions aimed at predictive diagnosis, since the electrical parameters I-V-P are perfectly separated in the part of the solar panel and in the part of the battery. Many of the most common faults and the cause of degradation in solar panels are manifested through a reduction in the power generated in the solar panel part. For this reason, when using MTTP regulators, supervision and parametric observation are carried out in the right place, and more precisely because the solar panel is the element susceptible to developing faults due to partial shading [11,16]. On the contrary, PWM regulators could mask and delay the detection of possible symptoms or small incipient faults that are developing in the solar panel part.

The predictive diagnosis method is applied specifically to avoid the phenomenon of degradation that leads to the development of hotspots, the appearance of which is random in nature and is caused by any type of occasional shading due to falling leaves, accumulation of dirt, cloudy conditions, bird droppings, manufacturing defects, etc., on the active face of the solar panel.

In addition, with the aim of achieving early isolation after the appearance of the predictive symptom of the possible fault, a decentralized regulation structure has been applied that was inspired by the design of distributed control systems and by the architecture adopted by the maximizing devices. The objective was to avoid the limitations that occur in the diagnosis of faults in structures centralized in a single regulator/inverter that make it difficult to detect and isolate faults in specific solar panels early. The decentralized structure also provides the advantage that in the worst case, if the fault occurs in the regulator, it will only affect that part controlled by the regulator. Nevertheless, in this case, it is advisable to ensure, due to its decentralized structure, the maximum functional synchronization of all MPPT charge regulators participating in the photovoltaic system.

This manuscript is organized as follows: In Section 2, sensors and devices used in the supervision of photovoltaic electrical energy are presented. The photovoltaic generation system used and the tests carried out are described in detail. The proposed method for PVI supervision and predictive diagnosis to avoid failure occurrences is explained. In Section 3, the results of the experiments are presented. In Section 4 contains a discussion of results as well as some additional considerations and findings obtained from the experimental trials. Finally, in Section 5, some conclusions regarding the advantages of the proposed method for fault prediction are drawn.

## 2. Materials and Methods

### 2.1. MPPT Regulator

The MPPT regulator is a type of solar regulator that incorporates a more complex technology than PWM regulators. Its operation is based on the search for the voltage point at which the panel offers the maximum possible power and operating in that condition until there is a change caused by partial shading, an increase in cloud cover, or temperature. Its efficiency is around 95% in terms of conversion while assuming a power gain that can reach 45% in winter and 15% in summer. Its main function is to correctly charge the batteries with the energy that comes from the solar panels, constantly control the state of the battery, and regulate the intensity of the charge to extend its useful life. Depending on the percentage of charge of the batteries, if it is below 95%, it will allow the passage of all the energy generated by the solar panels so that they are charged as quickly as possible. If the batteries are between 95% and 99% of their charging capacity (float state), the passage of energy will be very controlled to allow maximum charging. If the batteries are fully charged, it will interrupt the power supply to protect them from overheating and overloading. Therefore, the level of charge that the solar regulators must distribute will depend on the initial state of charge of the battery bank. With a solar charge regulator, the battery will always be protected against overloads and the charging is carried out when it is most convenient at any given time.

**Figure 4:**
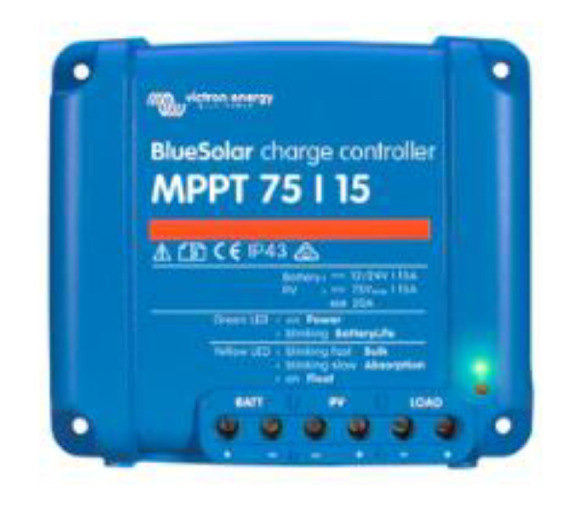
Victron BlueSolar MPPT Charge Controller.

The solar charger used in this work (Figure 4) used intelligent ultra-fast maximum power point tracking algorithms, which was especially beneficial when the intensity of sunlight was constantly changing, such as in cloudy conditions. Other conventional regulators, even though they use MPPT, usually select a local MPP that is not necessarily the optimal MPP. This type of ultra-fast MPPT controller collects 30% more energy than solar chargers with a PWM controller and up to 10% more than slower MPPT controllers [17].

**Figure 5:**
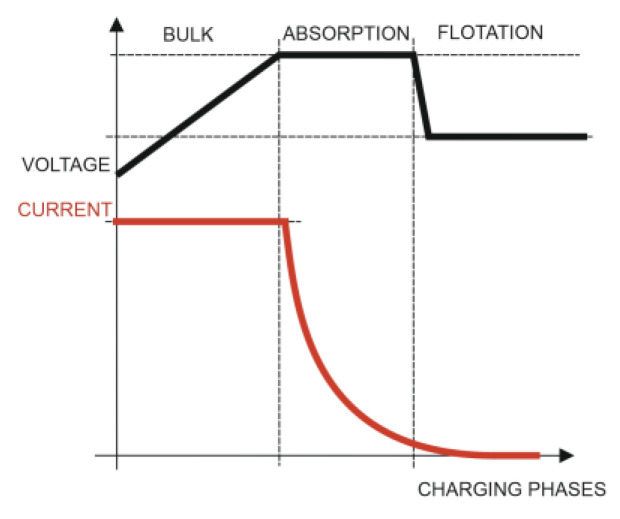
Batteries’ three-phase charge process.

The solar charger applied three charging phases (Figure 5):

- Initial charge: During the initial charge phase, the solar charger provided the maximum charge current to rapidly charge the batteries. During this phase, the battery voltage increased slowly. Once the battery voltage reached the set absorption voltage, the initial charging phase stopped and the absorption phase began.
- Absorption: During the absorption phase, the solar charger switched to constant voltage mode. The current flowing to the battery was gradually reduced. Once the current fell below 1A (tail current), the absorption phase stopped and the float phase began. When only surface discharges occurred, the absorption time was short. This prevented overcharging of the battery. After a deep discharge, the absorption time was automatically increased to ensure that the battery was fully charged.
- Float: During the float phase, the voltage was reduced and the batteries were fully charged.

The Victron Connect app allowed us to select from eight preset charging algorithms. The charging algorithm could also be fully programmed. The charging voltages, the duration of the phases, and the charging current could be adapted.

Some types of lead–acid batteries require periodic equalization charging. During equalization, the charging voltage will rise above normal charging voltages to balance the cells. If equalization charging is required, it can be enabled using applications such as Victron Connect.

The absorption and float charge voltages were adjusted according to the battery temperature, which is why a specific temperature sensor was required. Alternatively, the internal temperature of the solar charger could be used, which was necessary when charging lead–acid batteries in hot or cold weather conditions. Temperature compensation could be enabled or disabled in the solar charger settings. The amount of compensation could be adjusted by means of the compensation coefficient (mV/°C).

In cases in which greater accuracy is required, the use of an external battery temperature sensor should be considered. The temperature compensation range was 6 °C to 40 °C (39 °F to 104 °F). The solar controller’s internal temperature sensor was also used to detect if the solar charger had overheated. Ideally, a wireless battery voltage and temperature sensor should be considered that can be used in a complementary manner with the solar charger with the aim of compensating to improve charging efficiency and extend the life of lead–acid batteries. It also performs charging voltage compensation by increasing it in case there is a voltage drop across the battery cables when charging with a high current.

### 2.2. Synchronization of MPPT Charge Controllers with Cerbo GX

**Figure 6:**
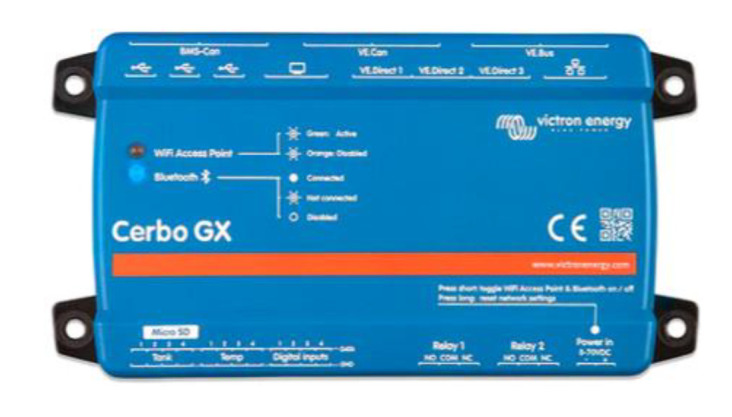
Cerbo GX Controller.

The Cerbo GX is a general control device for a photovoltaic system (Figure 6). The other components of the system such as solar regulators, inverters/chargers, supervision and monitoring devices, and batteries were connected to the Cerbo GX, which guaranteed the synchronized operation of the entire installation. The Cerbo GX can act as a passive element and also as an active element through which a maximum charge current of the entire network of charge controllers can be configured, such as the one used in this work. In case such a function was needed, the distributed current and voltage control (DVCC) [18] functions could be used. Additionally, the device allows firmware updates and configuration modifications remotely from anywhere using an Internet connection and through the Victron Remote Management portal. Cerbo GX is among the GX devices, which are modern tracking solutions from Victron running on the Venus OS operating system.

**Figure 7:**
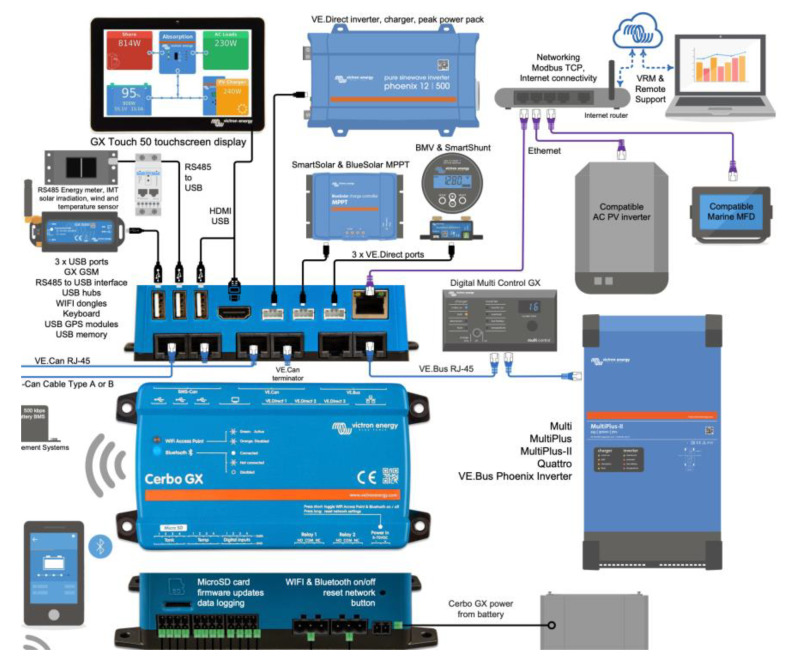
Cerbo GX configuration.

To ensure synchronization of the charge controllers, the Cerbo GX controller could be used in conjunction with four identically configured Smart Solar charge controllers. One possible configuration was to use a Victron Networking VE Smart network to charge the battery banks, which fulfilled the same mission as a single higher-power regulator. The decentralized solar chargers were synchronized using the same charging algorithm between them without the need for additional hardware and performed the bulk, absorption, and float charge state changes synchronously. Each charge regulator could manage its own output current, which depended mainly on the output of each photovoltaic field, the resistance of the cable, and the maximum output current configured in the charger, but sometimes it was convenient to configure a maximum load current of the entire network. In case this function was needed, the distributed current and voltage control functions (DVCC) [18] could be used. With this objective in this work, a Cerbo GX device was used (Figure 7).

There are certain types of systems in which synchronous charging is not necessary. This occurs in energy storage systems (ESSs) with managed batteries and in ESSs with unmanaged batteries that contain an inverter/charger that controls all of the solar chargers.

**Figure 8:**
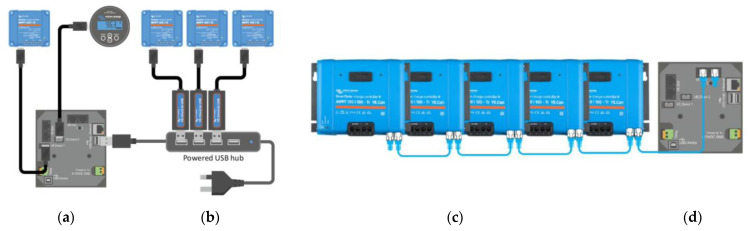
Two hardware connection alternatives for the synchronized operation of several MPPT controllers. (**a**) Cerbo GX (**b**) Powered USB hub (**c**) MTTPs serial network (**d**) Cerbo GX.

Two other alternative configurations for the synchronization of the chargers could be used:

- Connecting and synchronizing them through the VE Can inputs of the Cerbo GX. The charge controllers could be connected in series or as a daisy chain.
- Using a single USB input from Cerbo by using a powered USB hub connected to a controller such as a Cerbo GX so there was no need to pair them on a VE Smart network, which freed up other inputs that could be used for additional devices should system expansion be required (Figure 8).

The synchronization of the chargers was carried out through a type of primary–secondary system. The algorithm chose a primary charger among the set of existing solar chargers in the system, and that primary charger was the one that dictated the charging algorithm. However, all of the solar chargers that belonged to the same network had to be configured for the same type of batteries by the designer [19]. Then the primary will ensure that all the chargers are in the same state of charge and at the same voltage setpoint. As mentioned above, the battery-charging current was not controlled by the primary charger, but by each of the chargers individually. At the start of the day, the primary charger measured the battery voltage before any of the other chargers on the network began charging (to find the idle voltage of the battery). This information was used to decide what the total absorption time should be for some types of batteries. The idle voltage of the battery, as well as the total absorption time and the time spent in the current state of charge, was shared with the other chargers. That information was important because it allowed the chargers to resume the charging algorithm if for some reason the primary charger stopped charging (i.e., the sun went down, the charger failed, the charger lost contact with the grid, etc.).

**Figure 9:**
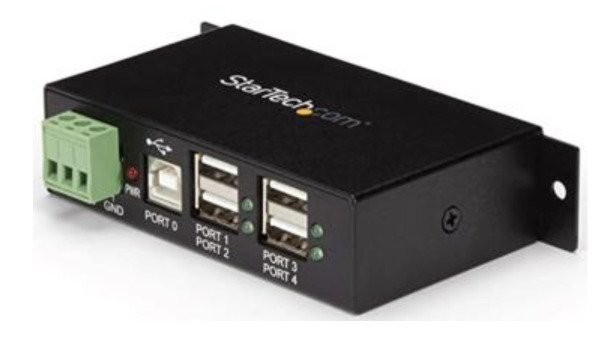
StarTech.com port hub.

In the absence of a specific battery current sensor, the network chargers combined their output current to estimate a better battery-charging current. This improved the accuracy of the tail current setting, a feature intended to finish the charging cycle sooner if necessary. In our system, to guarantee the synchronized operation of the charge regulators, a StarTech.com model [20] was used (Figure 9).

**Figure 10:**
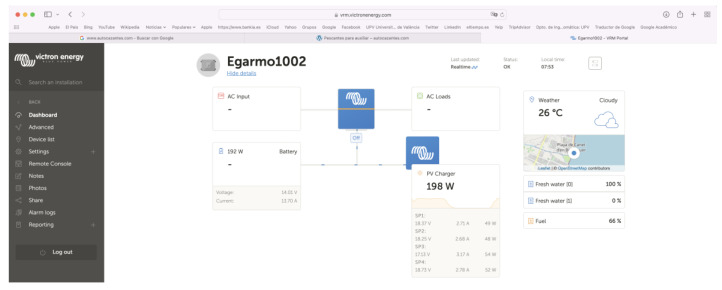
Victron connect app.

**Figure 11:**
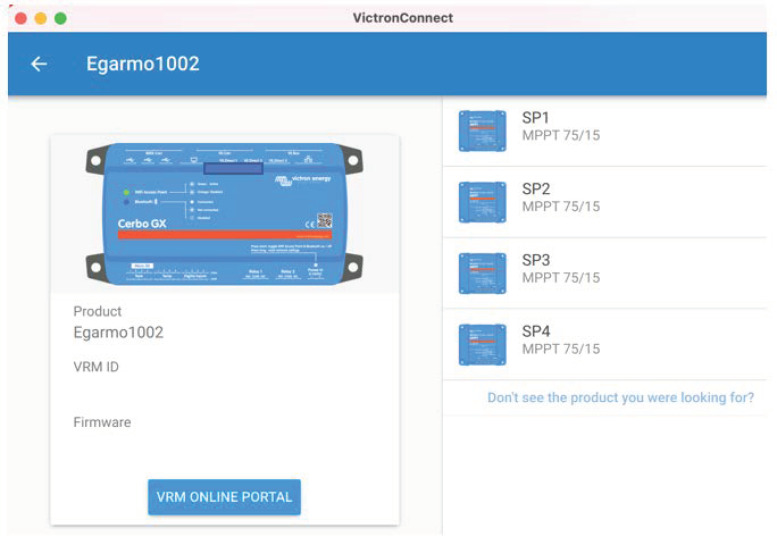
VRN online portal.

**Figure 12:**
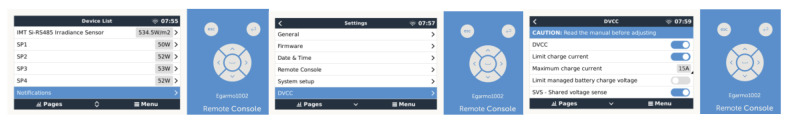
Synchronized operation of solar chargers using the DVCC function.

The objective of the Victron Connect App was to control, manage, configure, monitor, and update the software of the devices that were connected to it. We could access all available equipment parameters for easy and intuitive management to obtain real-time information as well as real-time and historical data from any Victron product via Bluetooth, USB, or WiFi/LAN/Internet through the GX device. Victron Connect works on iOS or Android phones and Windows or MacOS X laptops through an intuitive and simple interface [19,21]. To guarantee and configure the synchronized operation of the regulators with the Cerbo GX, the distributed current and voltage control (DVCC) functions were used (Figure 10, Figure 11 and Figure 12).

### 2.3. PV System Installation

The PVM chosen to carry out the experimental tests was from the Ecosolar company and had the characteristics shown in Table 1. It was a monocrystalline flexible panel. This option was chosen mainly because of its weight, adaptability to curved surfaces, and Wp/surface ratio.

**Figure 13:**
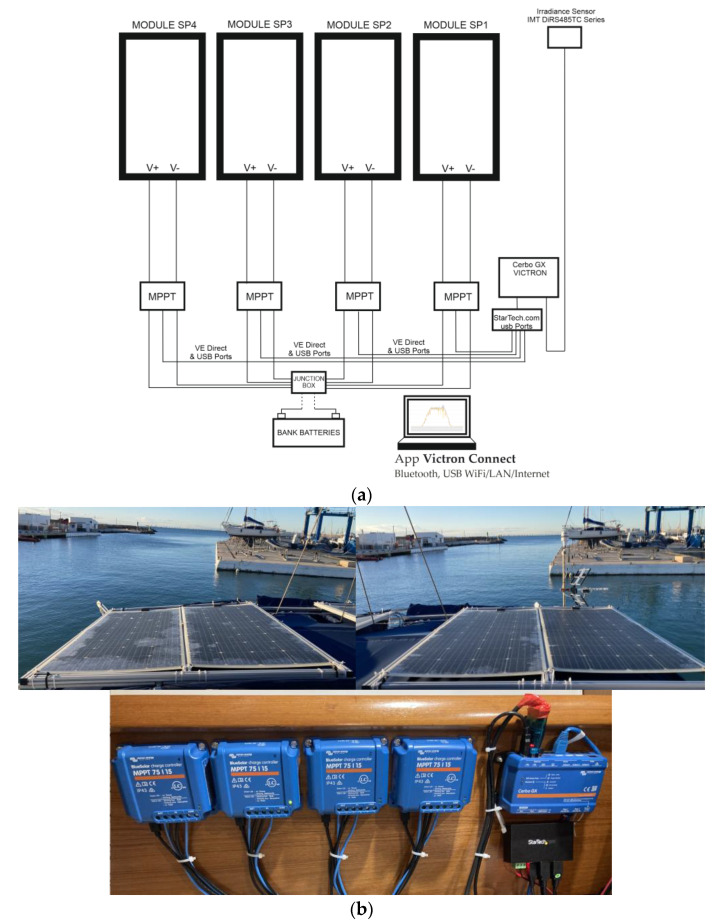
PV system installation: (**a**) schematic diagram; (**b**) actual assembly.

Four PVMs of the type and model described above were used in this experimental study. The four isolated panels were each connected in parallel through MPPT charge regulators. The outputs were connected to a common connection that fed a system of 12 V service batteries with a total load capacity of 750 Ah. Figure 13 shows the experimental test bench with the data acquisition system and associated sensor devices.

### 2.4. Diagnostic Algorithm

From the point of view of statistical process control (SPC) techniques, an industrial process is subject to a series of random factors that make it impossible for two production processes to work exactly the same. In the case of solar panels, even in initial conditions of use and immediately after being purchased from the manufacturer, they may present small differences in their performance; that is, they can present a certain variability in their operation even though they are being used under equal conditions. Therefore, the following fault causes were considered:

- Natural, random, or unassignable causes: due to chance, these were not identifiable and they could not be reduced or eliminated. They produced small variations.
- Assignable causes: identifiable and had to be eliminated. They produced large variations.

If the process is operating in such a way that there are small oscillations of all these factors but in such a way that none of them has a predominant effect over the others, then by virtue of the central limit theorem (CLT), it is probable that the quality characteristic of the manufactured product is distributed according to a normal law. The central limit theorem establishes that if a random variable is obtained as a sum of many independent causes with each of them being of little importance with respect to the set, then its distribution is asymptotically normal [22,23].

The fault diagnosis algorithm developed in this work was based on the concept of comparative trending analysis [24,25] of time series affected by seasonal and random components. The objective was to observe whether the quantitative values of the electrical parameter of the electrical power generated by each one of the sets of solar panels of the photovoltaic system were within a certain power generation range of small variability of a random nature, or in another case, if in any of the series there was a greater comparative deviation that was due to the occurrence of a specific assignable cause. In [26,27,28], techniques were used specifically for fault detection and diagnosis of photovoltaic systems based on statistical monitoring approaches; in [29], real-time fault detection in PV systems under MPPT was also applied.

Among the main assignable causes that produce a greater deviation are the partial shading phenomena that usually occur on one or more cells of a solar panel. The working hypothesis used for a small isolated photovoltaic system was that the disturbances derived from the differences in solar irradiance, irradiance plane, ambient temperature, wind intensity, and humidity would produce small energy-production differences between the panels. Therefore, these differences were random and not due to assignable causes such as reductions due to the shading phenomenon in the solar panels. For this reason, to carry out the comparative analysis, the standard element represented by the maximum value of the quantitative values systematically obtained at each instant of synchronized sampling obtained from each of the intervening series was used. The application of this algorithm in [11] was based on the hypothesis that the possibility that all the panels were partially shaded at the same time was very unlikely.

In this work, the algorithm for predictive diagnosis used to establish the predictor parameter was adapted to the most convenient observable and accessible parameter when using MPPT regulators.

The predictive diagnosis algorithm was based on the early detection of uncorrelated point deviations at each sampling event over the data series of the generated input power P_SPi_ to each MPPT.

The nature of deviations likely to be produced by the effects of the increasing degree of shading correlated with decreasing power deviations can be:

(a) TPD (total power decrease) due to the total activation of the bypass diodes;

(b) PPD (power percent decrease) due to quantized partial activation of bypass diodes;

(c) MTVPS (minimum threshold value of the predictive symptom) of P_SPi_ downward deviation for alert declaration observed during the application of the inductive shading method. This type of shading, which does not activate the bypass diodes, also does not prevent the degradation phenomenon. Consequently, it is the one that introduces the worst conditions regarding the degradation mechanism that continues to develop latently.

The behavior of the time series of the quantitative P_SPi_ values under normal conditions of power generation without shading showed a great correlation. In some cases, some of the series showed small or punctual deviations of a random nature individually, but the deviations did not persist. Partial shading caused constant and persistent comparative P_SPi_ deviations up to 12 W. This could be used as the predictive symptom for PS that signified that the shading phenomenon was taking place.

Therefore, the predictive algorithm for each of the point value samples applied the following search process:

Detect the P_SPi_ values of the set of measures in the current sample. Consider ${\left(P_{\mathrm{SPi}}t\right)}_{t=1}^{N};\left(P_{\mathrm{SP}}t:t=1,\ldots ,N\right),\;$ where P_SPi_t is the series sample observation number t (1 ≤ t ≤ N) for PVM number i for i ∈ {1,2, …, k} and N is the number of sample observations in the entire series (the length of the series). To apply comparative deviation values analysis in the same sampling time t, a set with the synchronized sampling t series power values of the k solar panels (SSCV) such as SSCV = {P_SP1_t,P_SP2_t,...,P_SPk_t} is needed. The N observations can be collected in a column vector SSCV = [P_SP1_t,P_SP2_t,...,P_SPk_t]′ of order N × 1.

Find the maximum value P_SPimax_t from the instantaneous quantitative series values of the current sample in the current vector SSCV. So, PSPimaxt ∈ P_SPimax_t ∈ {P_SP1_t, P_SP2_t,..., P_SPk_t}.

The comparative deviations analysis takes place among each of the SSCV elements and the maximum value MaxP_SPi_t = P_SPimax_t found in vector SSCV. Calculate the deviations of the other non-maximum series values from the maximum value PSPimax in the current sample DevP_SP_t = MaxP_SP_t − P_SPi_t.

Find deviations for which the condition is DevP_SP_t ≥ MTVPS. If the condition DevP_SPi_t ≥ MTVPS is true, it means that the SP_i_ solar panel is suffering a shadowing process.

Activate the alert for SP_i_ disconnection in the case of DevP_SPi_t ≥ MTVPS as a predictor symptom of shadowing occurrence on one or more solar panels.

**Figure 14:**
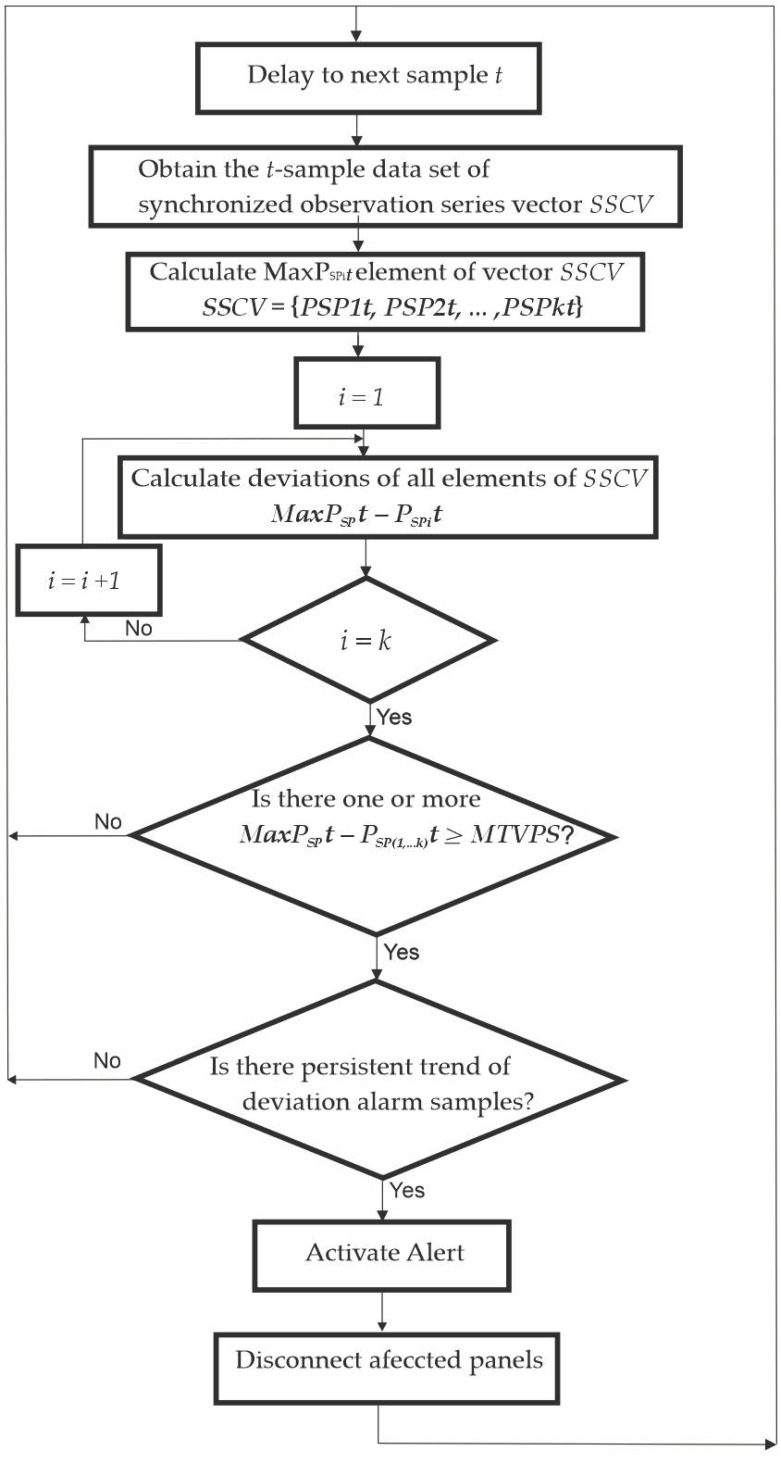
Online deviation trend comparative analysis algorithm flow chart.

Figure 14 shows the deviation trend comparative analysis algorithm flow chart.

## 3. Results

In a previous work [11], the partial shading fault inductive method was applied in the context of PWM regulators with a main objective of establishing the minimum threshold value of the predictive symptom (MTVPS) that could be used to diagnose faults. That method considered the restriction that the quantitative values of the I-V parameters would only be observable at the PWM output regulator in the battery part.

In the present work, an MPPT regulator was used for each of the four solar panels, which provided additional advantages both from the point of view of their performance as battery charge managers and from the point of view of their use in the application of predictive diagnosis techniques. The MPPT regulators allowed us to carry out the phases of early detection and isolation of faults specifically in each solar panel. Another additional advantage was that the use of an MPPT regulator for each panel made it possible to separate the solar panel part and the charging-battery part. In that way, the observation of the I-V-P parameters could be obtained in a specific manner that was more precise for the element susceptible to degradation.

**Figure 15:**
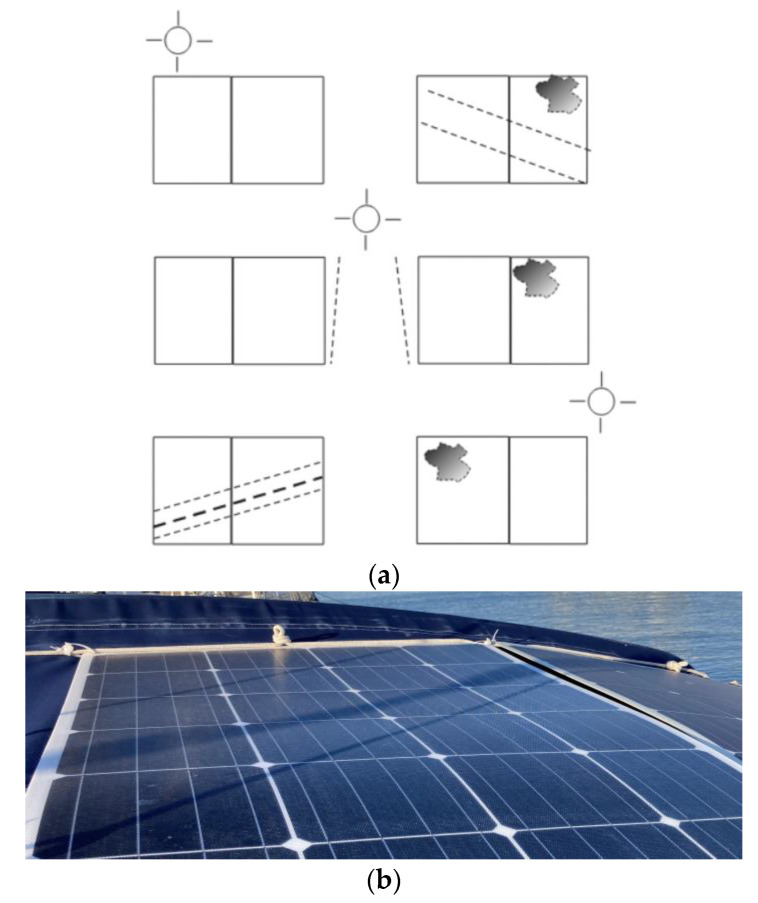
Displacement of the dashed lines and irregular shading areas according to the daily movement of the sun from east to southwest: (**a**) schematic diagram; (**b**) actual assembly.

**Figure 16:**
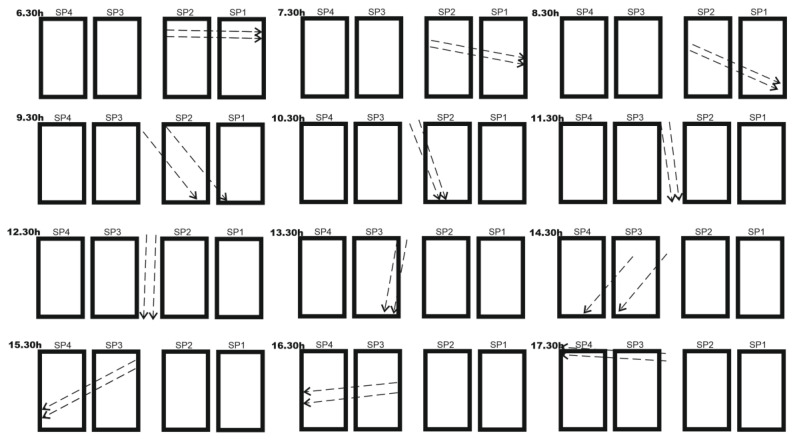
Daily partial shading displacement by the hour.

With the observation of the effects of partial shading on the photovoltaic installation, a fixed scenario was established, as shown in Figure 15 and Figure 16. In Figure 15a, the projection of the shadows is represented in a simplified way, where the thicker dashed line and the irregular shape that represent the projection of the mast and the antenna of a meteorological station are included. Figure 15b shows the actual assembly. This controlled scenario allowed us to establish correlation among where, when, and why a decrease in the power generated in each PVM would be produced during the day.

The test was carried out last 30 April from 6:30 a.m. to 5:30 p.m. (UTC), during which the shading projections that were gradually produced on the specific solar panels during the day were described. At the beginning of the morning from 6:30 a.m. to 9:30 a.m., two shading lines were projected toward the SP1 and SP2 solar panels. At 9:30 a.m., the two shadow lines began to affect only the SP2 solar panel. Between 11:30 a.m. and 12:30 p.m., the shadow projections remained in no man’s land without affecting any of the solar panels. In the afternoon at 1:30 p.m., the two shadow lines were projected toward the SP3 solar panel only. From 2:30 p.m. to 5:30 p.m., the shadows were projected toward the SP3 and SP4 solar panels at projection angles that changed gradually as the sun moved to the southwest (Figure 16).

The measurements were taken to carry out a comparative trend analysis of the set of parametric series of the generated power representative of the performance of each of the four solar panels. The comparative analysis was carried out systematically online for each of the synchronized samples of each of the four intervening series. In order to be able to make the best possible comparison with the analysis carried out in [11], the same positioning of the solar panels was maintained with respect to the displacement of the sun while trying to find the same shading effects on the panels.

**Figure 17:**
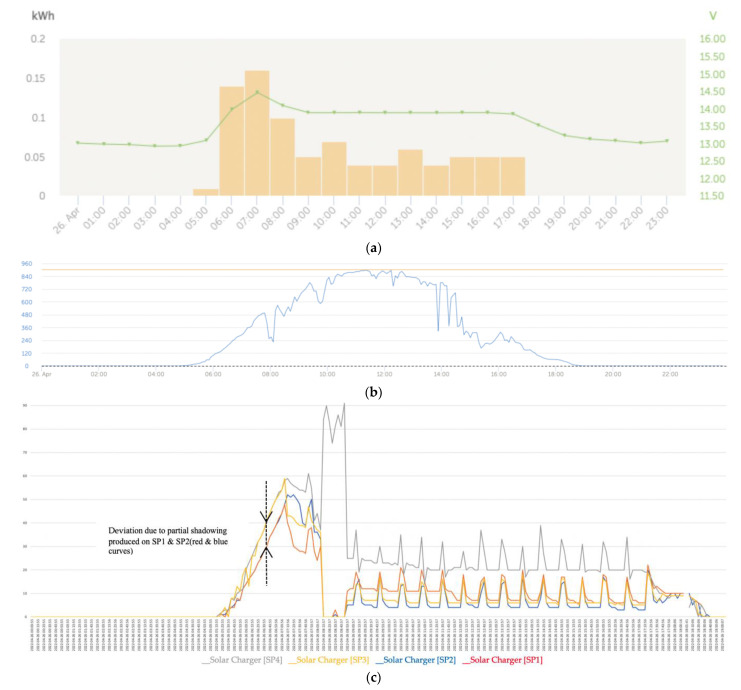
(**a**) Daily charge phases: initial, absorption, and flotation (26 April). (**b**) Solar irradiance in W/m^2^ (26 April). (**c**) Deviation trend comparative PV power data series analysis due to partial shading in watts (26 April).

The graphs that follow show the daily battery bank charge graphs with the development of the three fundamental phases that characterized the charging, which included: (1) initial charge (bulk); (2) absorption; and (3) float of the batteries (Figure 17a). The associated graphs of the solar irradiance level of that same day that are shown in Figure 17b considered the importance of its correlation with the levels of partial shading on the solar panels. Finally, the four series of the power generated in the four solar panels of the installation are shown in Figure 17c.

One of the additional capabilities of the type of MPPT regulator used is the application of different types of intelligent charging algorithms, as was the case of that used in this work, which had up to eight different algorithms specially dedicated to safeguarding the batteries through the application of intelligent management methods with the aim of avoiding battery overload combined with the operation from the maximum power point of those panels suffering from partial shading.

The shape of the set of curves of the series was totally influenced by the three-phase charging algorithm developed for the four MPPT solar regulators. In Figure 17a (voltage), the initial charge (bulk) (5:13 a.m.), absorption (7:13 a.m.), and float (8:13 a.m.) phases of the batteries were applied synchronously by the solar regulators on the battery bank. Figure 17c shows the four power series generated by the solar panels SP1, SP2, SP3, and SP4 installed in the configuration shown in Figure 16.

At the beginning of the day in the left lateral band, there was a trend of reduction in the curves that corresponded to the power produced in the solar panels SP1 and SP2 with respect to SP3 and SP4 due to the partial shading that was being projected on the first panels.

**Figure 18:**
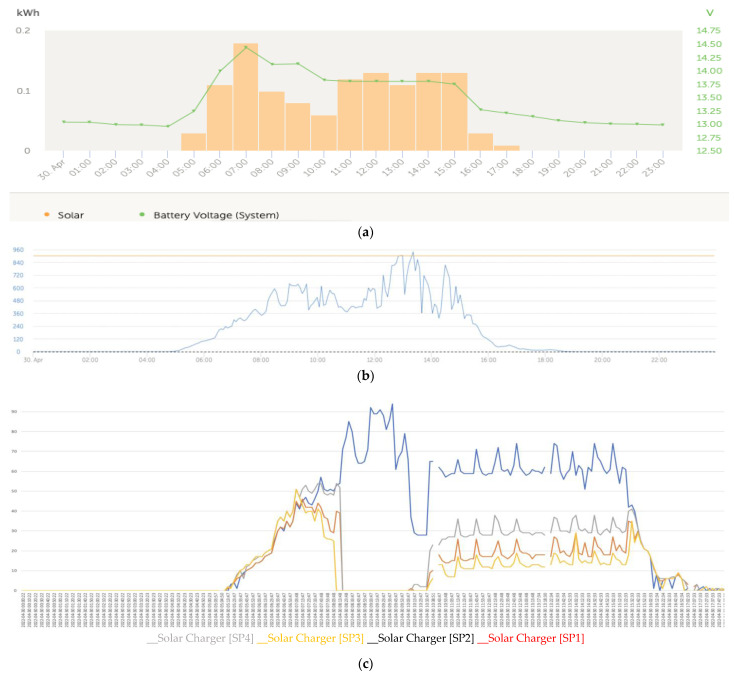
(**a**) Daily charge phases: initial, absorption, and flotation (30 April). (**b**) Solar irradiance (30 April). (**c**) Deviation trend comparative PV power data series analysis due to partial shading (30 April).

As shown in Figure 18c, after the bulk charge phase, the charge regulators reduced the battery voltage by decreasing the charge current and maintained the batteries’ voltage at the predetermined voltage value for the float charge stage. In this stage, the batteries were charged by synchronously emitting small spikes at a certain frequency in a kind of trickle regime to ensure that the batteries remained fully charged.

**Figure 19:**
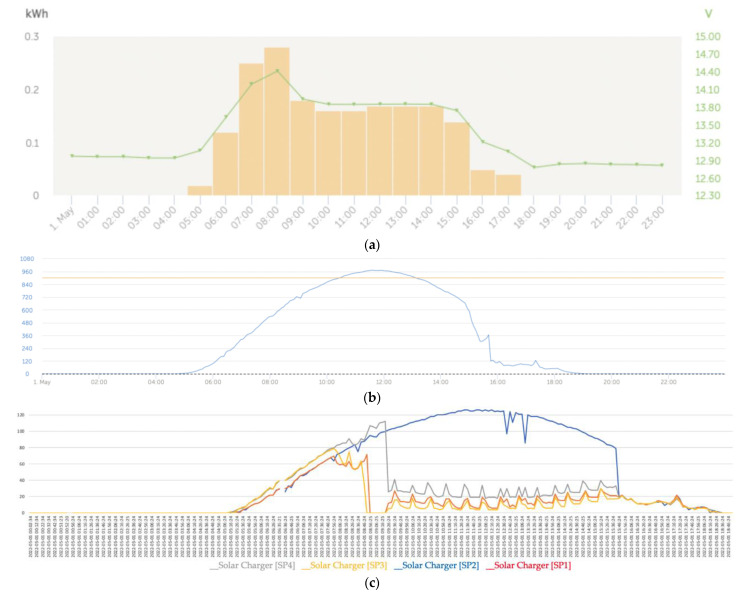
(**a**) Daily charge phases: initial, absorption, and flotation (1 May). (**b**) Solar irradiance (1 May). (**c**) Deviation trend comparative PV power data series analysis due to partial shading (1 May).

Figure 19a shows that regarding the battery charging, the initial charge (bulk) began at 5 a.m. and ended at around 8:30 a.m. followed by the absorption phase. Figure 19c shows that at the beginning of the day, the power generation series presented a trend of the curves of the power series that adjusted to the effects of the reduction in the power produced in the solar panels SP1 and SP2 due to the shading being cast on those panels. On the contrary, at the end of the afternoon, the four series converged due to the increased cloudiness produced, which is why the partial shading effect that took place on SP3 and SP4 was blurred.

On the other hand, in this case, we observed that the algorithm of the solar regulator associated with the solar panel SP2 tended to favor the generation of the maximum power because it was the panel that was experiencing the shading with the maximum intensity.

At the beginning of the day until approximately 7:46 a.m., the behavior of the trends of the SP1 and SP2 series was expected because they were experiencing the effects of partial shading with a tendency to reduce power compared to the SP3 and SP4 panels, which, during the first hours of the morning, did not experience any type of partial shading.

The intelligent distributed battery-management system formed by the Cerbo GX and the Victron BlueSolar MPPT charge controller prevented the batteries from overcharging, which was combined with the operation from the maximum power point of those panels suffering from partial shading, as can be seen for the SP2 panel in Figure 19c. However, from that moment on, the capacity of the MPPT solar regulator intervened to cause the SP2 solar panel to operate at the maximum power, which can be seen in Figure 16 because it was the one that was experiencing the shading phenomenon with the greatest intensity. This action implicitly carried out a balance of lower energy contribution by the remaining solar panels because one of the regulator’s missions was to protect the batteries from some type of overload. In general, we observed that the dynamics of trend changes in the power series were determined by the changes that occurred in the series of solar irradiance.

**Figure 20:**
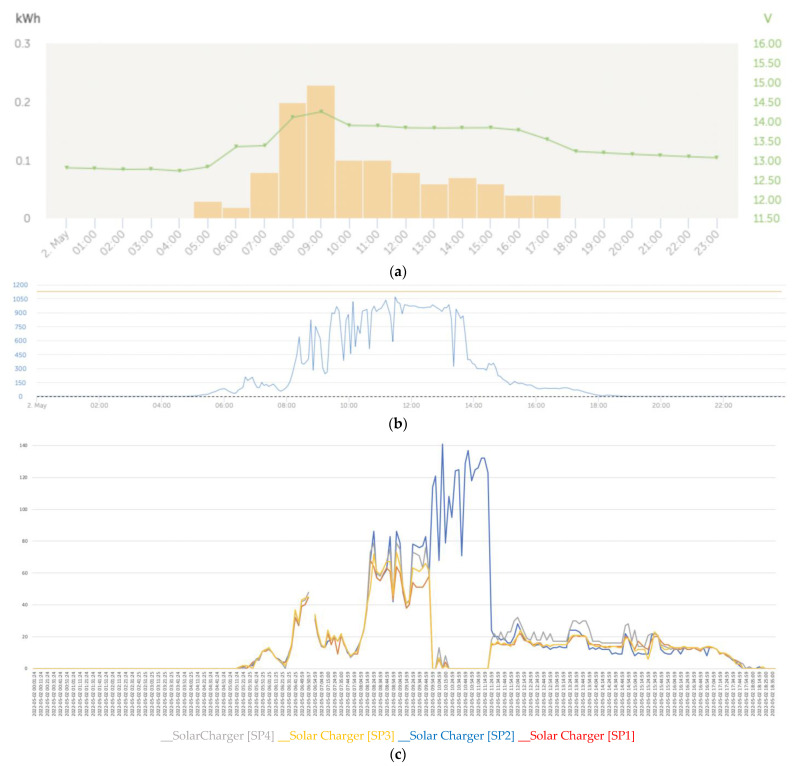
(**a**) Daily charge phases: initial, absorption, and flotation (2 May). (**b**) Irradiance (2 May). (**c**) Deviation trend comparative PV power data series analysis due to partial shading (2 May).

Figure 20a shows the effects on the series due to a great variability in the solar irradiance levels (Figure 20b). At the beginning and end of the day, which correspond to the left and right lateral bands, the series converged notably (Figure 20c), unlike the central band in which areas of certain divergence developed between the series.

**Figure 21:**
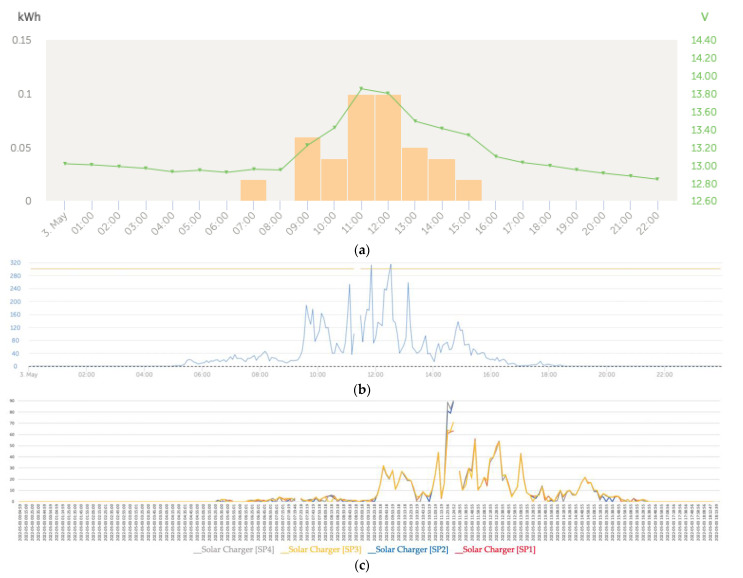
(**a**) Daily charge phases: initial, absorption, and flotation (3 May). (**b**) Solar irradiance (3 May). (**c**) Dense cloudiness blurred the shading effect, causing the convergence of the series (3 May).

The graphs in Figure 21 were obtained during a very cloudy day accompanied by heavy rains and characterized by a strong decrease in solar irradiance. As shown in Figure 21c, the effects of heavy cloud cover caused the shading to blur, resulting in the power curves tending to converge.

**Figure 22:**
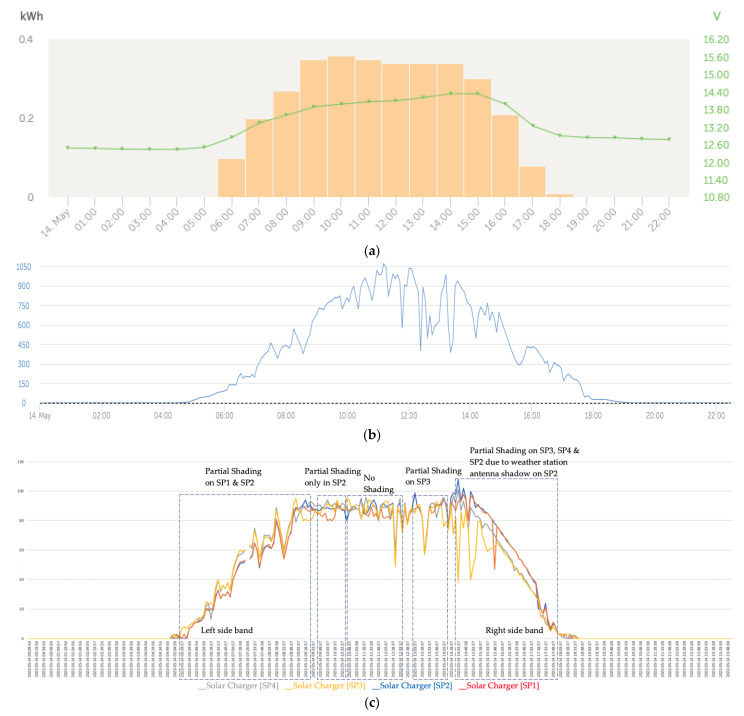
(**a**) Daily charge phases: initial, absorption, and flotation (14 May). (**b**) Solar Irradiance (14 May). (**c**) Deviation Trend Comparative PV Power Data Series Analysis (14 May).

Figure 22c shows a characteristic shape of the power series that is repeated assiduously in each of the sidebands of the graphs. This form of the power series without the appearance of the phase of small load peaks was due to the fact that in cases in which there was a strong demand for load consumption, it caused the float phase not to occur, passing from the absorption phase back to the bulk phase. In the left lateral band of the graphs, due to the situation of the sun and the projection of the shadows that affected the solar panels SP1 and SP2 (red and blue colors), the power-reduction deviations affected the solar panels SP1 and SP2. The quantitative values of the generated powers can be verified in Table A1 in Appendix A. We verified that the columns of the shaded series converged and the non-shaded ones also converged but between the two groups, there was a notable deviation. In the afternoon when the sun was located to the west, on the right lateral band the projection of the shadow lines was placed first on the SP3 solar panel and later on the SP3 and SP4 panels, corresponding to the grey and yellow curves located to the east, which meant that in the western sideband, the direction of the deviation of the powers was inverted in favor of SP1 and SP2.

Figure 22a shows the graph of the charge stages. Sometimes the load required almost all of the solar energy generated. If the load consumed all the energy provided by the system, the regulator could not maintain the battery voltage in the float stage. When the battery voltage equaled the preset value to improve recovery charging, the system exited the float-charge stage and re-enters the fast-charge stage or even returned to the bulk phase from the absorption phase without going through the float phase, as in the case shown in Figure 22a.

As an example of the deviation with a reduction in the generated power that occurred correlated with the shading that was projected on the SP3 and SP4 panels, Table A3 in Appendix A shows a part of the quantitative values obtained from the sampling process carried out by the Cerbo GX controller from 15:08:37 to 17:13:38 of the corresponding graph in Figure 22c made on 14 May 2022. In Table A3, referring to the right lateral band of the power series, a comparative analysis of the values generated in the two groups of series could be carried out between the group of the two panels that did not experience partial shading (SP1 and SP2). The deviation that occurred between both series of that group was negligible; on the other hand the deviation that occurred between the two panels that did experience shading (SP4 and SP3) was also negligible, but between the two groups ((SP1 and SP2) and (SP4 and SP3)), there was a notable deviation.

**Figure 23:**
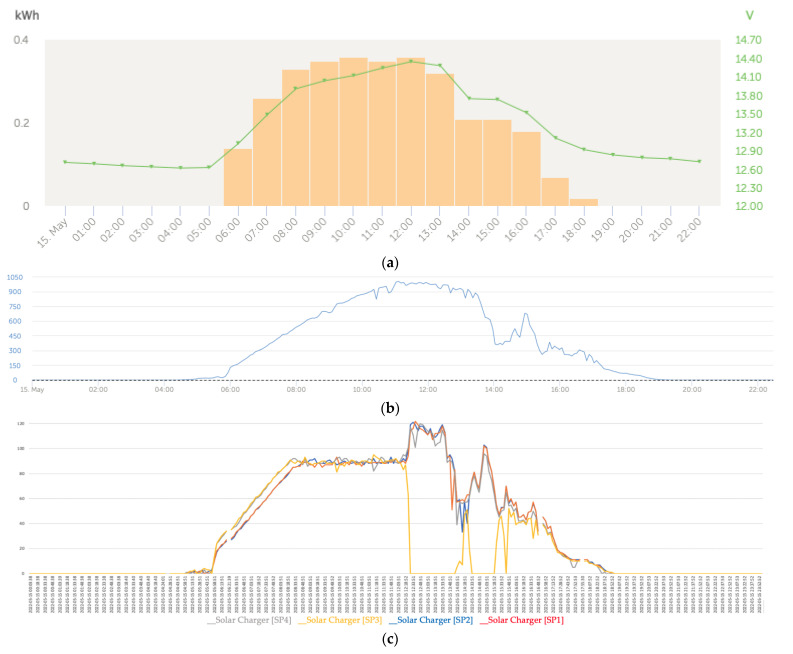

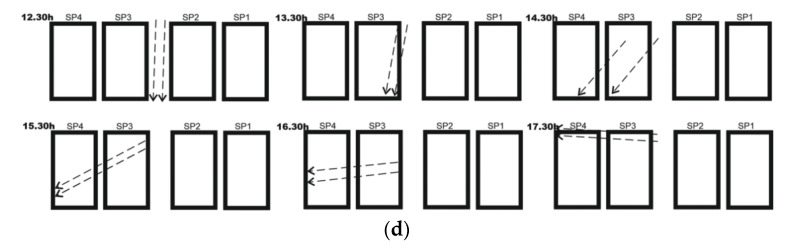
(**a**) Daily charge phases: initial, absorption, and flotation (15 May). (**b**) Irradiance (15 May). (**c**) Deviation Trend Comparative PV Power Data Series Analysis (15 May). (**d**) Daily partial shading displacement by the hour.

Figure 23c shows the effect of the high level of irradiance that existed that triggered the two bypass diodes in SP3, for which partial shading occurred from approximately 12:00 to 15:18. However, even though partial shading remained until 5:30 p.m., the firing of the bypass diodes was interrupted around 3:40 p.m. as the solar irradiance levels decreased. From that moment on, the curves of the series corresponding to the SP3 and SP4 solar panels showed a downward deviation with respect to the curves of the SP1 and SP2 solar panels because the shadows were projected toward the SP3 and SP4 panels.

On the other hand, we observed that when the yellow curve corresponding to the SP3 solar panel series fell to zero as a result of the firing of its two bypass diodes, the intelligent control algorithm of the solar regulator acted by increasing the energy supply of the three other solar panels to compensate for the contribution made by SP3. We verified that a high level of solar irradiance favored the tripping of the bypass diodes in the case of partial shading.

**Figure 24:**
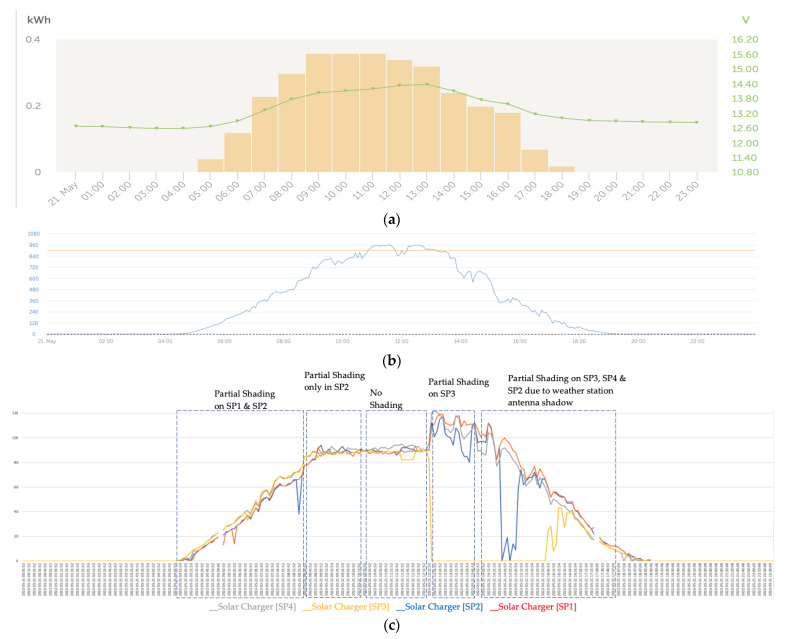
(**a**) Daily charge phases: initial, absorption, and flotation (21 May). (**b**) Irradiance (21 May). (**c**) Deviation trend comparative PV power data series analysis due to partial shading (21 May).

**Figure 25:**
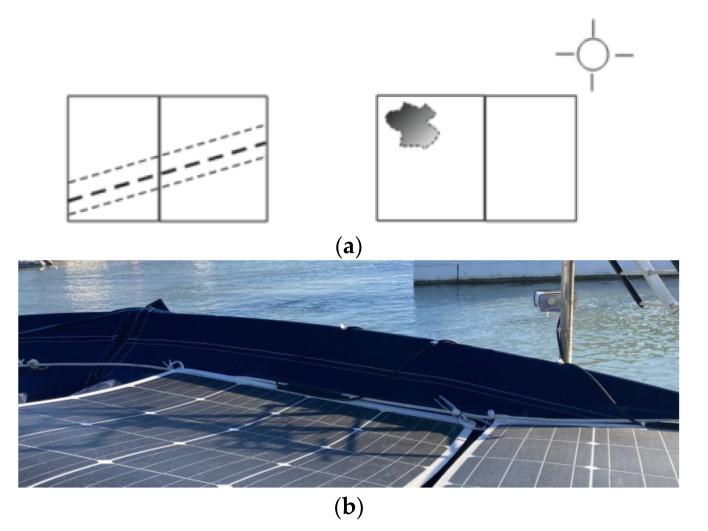
Shading of a weather station antenna: (**a**) simplified diagram; (**b**) actual assembly.

Figure 24 shows the tripping of the bypass diodes in the solar panel SP3 and various unstable trippings of SP2. In the case of the latter, the unstable shots were due to the fact that it was affected by the shading of a weather station antenna installed further to the southwest than the backstay cables (Figure 25).

## 4. Discussion

In fault diagnosis applied to the analysis of photovoltaic installations (PVIs), numerous works have been used based on the application of neuron network (NN) models both as a comparison pattern for their diagnosis and to make estimates of their performance. However, despite the attractive interest in the use of artificial intelligence techniques based on NNs, comparisons have been made between analytical models and those based on NNs in which the precision obtained was in favor of the analytical models [30]. In any case, both types of models share the problem that they do not easily incorporate the changing and adaptive nature of the natural, random, and multifactorial degradation factors that should be considered throughout the useful period of performance, especially in installations for which operation takes place in outdoor conditions, such as photovoltaic installations.

In [31], a relatively recent review of the literature highlighting challenges, current approaches, and opportunities for PV predictive maintenance was carried out. In this work, a cost-versus-detection-accuracy comparison was made. It compared: (1) manual diagnostics; (2) FMEA approaches; (3) machine learning and forecasting; and (4) real-time sensors as detection methods. This study concluded that (1) was the least expensive; on the other hand, (4) was the most expensive option but offered the highest detection accuracy. The paper concluded with a call to action to establish a collaborative agenda for prioritizing PV predictive maintenance.

In [26], residuals arrays of current, voltage, and power using measured temperature and irradiance were generated to capture the differences between the measurements and the predictions. Then, a multivariate exponentially weighted moving average (MEWMA) statistical monitoring chart of the residuals was used to detect faults. A similar method was used in [28].

In [27], different statistical diagnostic methods were presented:

- A method that provided operating parameters that were not sufficient to evaluate plant performance and detect failures; therefore, a daily corrected performance ratio that considered weather conditions was evaluated.
- Another method used outlier detection rules that did not require weather data or model training with detection methods such as 3-sigma, a Hampel identifier, and a box plot to identify PV string normal operation based on individual string current measurements.
- A third method used a monitoring system based on a power loss analysis using statistical signal processing to be compared with another one obtained in a MATLAB/Simulink environment in real time. Based on this comparison, a residual signal was generated. Then, a Wald test was applied to this signal in order to detect alarm signals from data captured randomly and consequently facilitate the decision making.

In [29], a lab-implemented typical grid-connected PV system was used to validate the fault-diagnosis performance of data-driven methods against real faults under MPPT/IPPT modes and practical conditions. In this work, statistical methods incorporated the general knowledge of the system’s functionality in order to construct a reliable and effective fault-diagnosis algorithm.

The fundamental differences between our method and the procedures used in previous references was that our system was particularly suitable for isolated “real world” installations, the characteristics of which allow a comparative trend analysis of all the power generated by each of the solar panels in online condition monitoring. Subsequently, for the assertion and adequacy of the type of predictive symptom associated with hotspot failures, a criterion based on statistical process-control methods was applied in order to assert that the predictor symptom occurred in a sustained and persistent manner over time due to an assignable cause and not a random cause.

In [11], to carry out the tests, PWM-type regulators were used and the observations made were in the battery part. As explained in the Introduction, in this type of regulator there is no functional separation between the solar panel and the battery. Then, the voltage parameter is established by the battery and not the output voltage of the panel, where the decrease in power due to the partial shading can be precisely appreciated. In the case of MPPT-type charge regulators, there is a functional separation that allows the specific electrical parameters of the solar panel to be observed in the regulator. Therefore, when using MTTP regulators, supervision and parametric observation is carried out in the right place and more accurately because the solar panel is the element susceptible to developing faults due to partial shading.

The application of the method of comparative analysis of the time series of the power curves generated by the solar panels showed that the existence of the phenomenon of partial shading of specific solar panels could be detected and isolated in an early predictive manner. This detection could be done using the persistent comparative deviation of the decrease in power between the PVMs as a predictive symptom that the cause of the degradation that leads over time to the development of hotspots is occurring.

In our general test, we verified that the series showed a convergence and also a decreasing trend that occurred between pairs that depended on whether they experienced shading or not. Panels throughout the day experienced shaded and unshaded states that changed based on their position and the path of the sun over the course of the day. As can be seen graphically in Figure 22c and numerically in Table A1, Table A2 and Table A3 in Appendix A, when there were causes assignable to the effect of partial shading, the deviations in the series corresponding to the affected solar panels presented notable deviations in power reduction alternately in each of the sidebands. This phenomenon can be seen especially in the data in Table A1 and Table A3 in Appendix A.

However, it is very important to dispel the doubt that the deviations were random, which could be resolved through the analysis of the persistence of the deviations with prolonged periods of affectation. Therefore, regarding the concept of amplitude and range of the deviation, it was necessary to add the criterion that the deviation was persistent in order to reliably affirm that there were predictive symptoms that the degrading phenomenon leading to the development of hotspots was taking place.

The shadow projection model in Figure 16 was a still photo obtained April 2022 (the model of course will experience certain variations due to changes in the path of the sun throughout the year). In the period in which partial shading effects did not occur, as occurred approximately between 11:30 a.m. and 1:30 p.m. of the obtained fixed model (Figure 16, Figure 22c, and Table A2 in Appendix A), the deviations that occurred were of a random nature and in general they presented a smaller deviation; although some deviations were punctually of greater amplitude, they were not persistent, so based on this behavior, we concluded that partial shading did not occur.

In this work, a new criterion was used to establish the selection of the predictive symptom as the MTVPS threshold value (minimum threshold value of the predictive symptom) with greater precision. In [11], the threshold value was obtained from the induced shading method applied to a set of solar cells of one of the PVMs. In our study, to obtain the MTVPS, the comparative value obtained from the effects of the power reduction produced by the shading of an 8 mm diameter steel cable in the record made on 14 May 2022 was used. These data are reflected in Table A1, Table A2 and Table A3 in Appendix A. This analysis allowed us to affirm that the predictor symptom could be quantified in a range of deviation between 5 and 10 watts with respect to the verification parameter DevP_SP_t = MaxP_SP_t − P_SPi_t.

The corresponding threshold values in the shading phases were obtained from the data collected in the two lateral bands shown in Figure 22c, in which the lack of convergence between the pairs of series (SP1, SP2) on the one hand and (SP3, SP4) on the other is clearly reflected. In addition, between these pairs of series, the mutual exclusion condition was observed; that is, when the pair (SP1, SP2) was shaded, (SP3, SP4) was not, and vice versa.

The effects of intense solar irradiance are very decisive on the effects of partial shading and its negative effects in the possible development of hotspots. Intense cloudy climatic conditions tended to decrease the intensity of solar irradiance so the shading was blurred; consequently, all the power series tended to converge more closely. As solar irradiance levels rose, the contrast of the comparative deviation of the series due to shading was more noticeable, as well as the effect of power reduction in the affected solar panels.

In our method, no additional external sensors were necessary because the sensors that measured the electrical parameters of the solar module part and the battery part were inherent to the type of MPPT regulator used. The decentralized system of charge regulators also did not imply any increase in system costs. Quite to the contrary, a charge regulator equivalent to the sum of the total power to be regulated supposes a higher cost.

**Figure 26:**
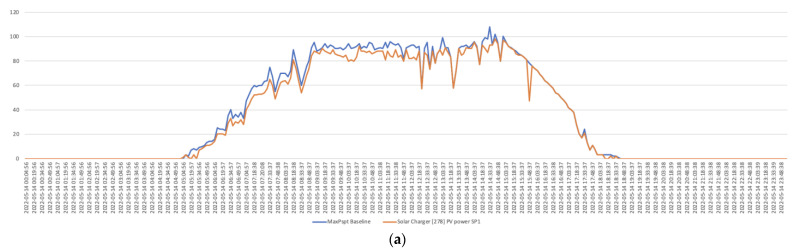

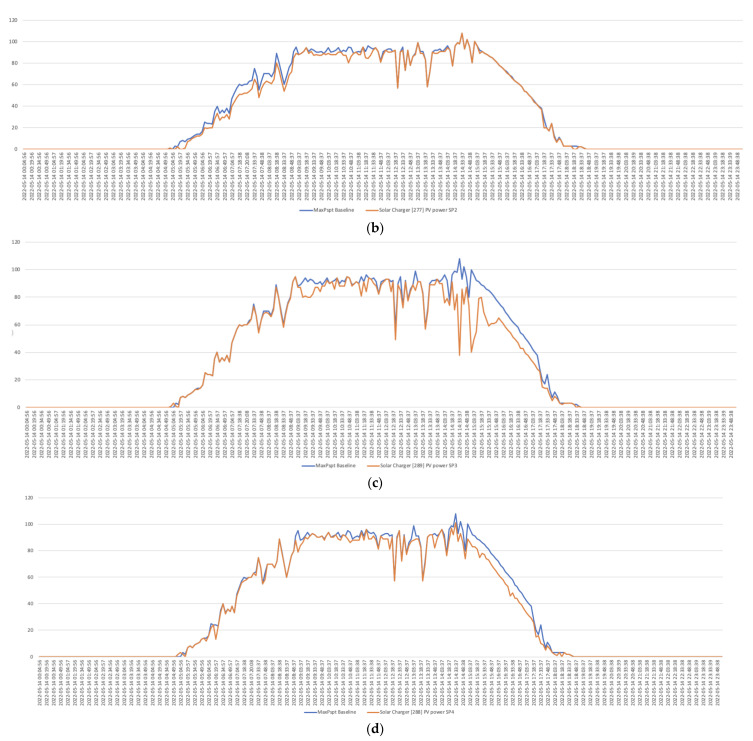
Comparative analysis of the baseline obtained from each of the MaxPspit values and the specific values of the power generated in each solar panel: (**a**) SP1; (**b**) SP2; (**c**) SP3; (**d**) SP4.

Finally, Figure 26 presents a comparative analysis between a series of a basal curves formed by the values of each MaxPspit at each sample time and the quantitative values of the individual series of the power generated in each module (SP1, SP2, SP3, and SP4). The figure shows, in a very intuitive way, those persistent (non-random) deviations in power reduction as predictive symptoms assignable to the effect of partial shading in each of the solar modules.

## 5. Conclusions

Partial shading caused constant and persistent comparative P_SPi_ quantifiable deviations that could be used as the predictive symptom that the shading phenomenon was taking place.

The use of MPPT regulators allowed us to observe the predictive electrical parameters I-V-P of the solar panel part that were carried out in a specific way. This was an important advantage over PWM regulators, for which the observation of these parameters can only be carried out in the battery part, limiting the observation only to parameter I of the output current of the PWM regulator.

The use of individual MPPT charge regulators in each of the intervening solar panels, which was contrary to the custom of using a single regulator in the solar installation, provided an action similar to that carried out by the maximizing devices that acted individually on each of the solar panels. This fact allowed us to develop predictive diagnostic techniques specifically for each of the solar panels, guaranteeing the early detection and isolation of the predictive symptoms of the degrading phenomenon that led to the inevitable development of hotspots. Additionally, the MPPT regulators used had more sophisticated charging algorithms with the objective of safeguarding and prolonging the useful life of the battery bank.

## Figures and Tables

**Table 1:** PVM technical characteristics.

Peak Power (P_max_)	(W)	160 ^1^
Production tolerance	(%)	±3
Maximum power current (I_mp_)	(A)	8.88
Maximum power voltage (V_mp_)	(V)	18.0
Short circuit current (I_sc_)	(A)	9.59
Open circuit voltage (V_oc_)	(V)	21.6
Weight	(Kg)	2.7
Dimensions	(mm)	670 × 1510 × 3
Maximum system voltage	(VDC)	500
Cell technology	Type	Monocrys
Cell brand	Name	Ecosolar

^1^ All technical data in standard test conditions. AM = 1.5 E = 1000W/m^2^ Tc = 25 °C.

## Appendix A

The following tables show the quantitative values (W) of the four series and deviations taken at a frequency of 5 min.

**Table A1:** SP quantitative power values and deviation registered the 14 May 2022 from 06:34:57 to 09:03:37 hours.

Time Stamp UTC(+00:00)	Charge State	SP4 Power	SP3 Power	SP2 Power	SP1 Power	P_SPimax_t Deviation	Partial Shadow
14 May 2022 06:34:57	Bulk	40	40	33	33	7	Partial shadow on SP1 and SP2 based on Figure 16. Partial Shadow on SP1 & SP2 Based on Figure 16
14 May 2022 06:39:57	Bulk	32	33	27	27	6	
14 May 2022 06:44:57	Bulk	36	36	30	30	6	
14 May 2022 06:49:57	Bulk	34	34	29	29	5	
14 May 2022 06:54:57	Bulk	38	38	32	32	6	
14 May 2022 06:59:57	Bulk	33	33	28	28	5	
14 May 2022 07:04:57	Bulk	46	47	40	40	7	
14 May 2022 07:09:57	Bulk	51	52	44	44	8	
14 May 2022 07:14:57	Bulk	56	57	48	49	9	
14 May 2022 07:18:38	Bulk	57	60	51	52	9	
14 May 2022 07:18:47	Bulk	58	59	51	52	8	
14 May 2022 07:20:03	Bulk	60	60	52	53	8	
14 May 2022 07:20:08	Bulk	61	61	52	53	9	
14 May 2022 07:23:37	Bulk	63	62	54	54	9	
14 May 2022 07:28:37	Bulk	61	64	56	57	8	
14 May 2022 07:33:37	Bulk	75	73	65	65	10	
14 May 2022 07:38:37	Bulk	67	67	60	60	7	
14 May 2022 07:43:37	Bulk	55	54	48	49	7	
14 May 2022 07:48:38	Bulk	58	63	56	56	7	
14 May 2022 07:53:37	Bulk	70	68	61	62	9	
14 May 2022 07:58:37	Bulk	70	69	63	63	7	
14 May 2022 08:03:37	Bulk	70	68	62	64	8	
14 May 2022 08:08:38	Bulk	67	66	61	61	6	
14 May 2022 08:13:37	Bulk	72	71	65	66	7	
14 May 2022 08:18:38	Bulk	89	87	80	81	9	
14 May 2022 08:23:37	Bulk	79	80	73	74	7	
14 May 2022 08:28:38	Bulk	70	68	63	63	7	
14 May 2022 08:33:37	Bulk	60	58	54	54	6	
14 May 2022 08:38:37	Bulk	68	67	61	61	7	
14 May 2022 08:43:37	Bulk	76	75	69	69	7	
14 May 2022 08:48:37	Bulk	80	79	72	73	8	
14 May 2022 08:53:38	Bulk	88	91	85	84	7	
14 May 2022 08:58:37	Bulk	79	95	89	88	7	
14 May 2022 09:03:37	Bulk	84	87	88	87	4	

**Table A2:** SP quantitative power values and deviation registered the 14 May 2022 from 09:03:37 to 17:13:38 hours.

Time Stamp UTC(+00:00)	Charge State	SP4 Power	SP3 Power	SP2 Power	SP1 Power	PSPimaxt Deviation	Partial Shadow
14 May 2022 09:03:37	Bulk	84	87	88	87	4	Partial shadow on SP1 and SP2 based on Figure 16.
14 May 2022 09:08:37	Bulk	86	87	89	86	3	
14 May 2022 09:13:37	Bulk	90	80	91	90	11	
14 May 2022 09:18:37	Bulk	89	81	94	88	13	
14 May 2022 09:23:37	Bulk	91	80	89	87	2	
14 May 2022 09:28:37	Bulk	93	80	91	86	13	
14 May 2022 09:33:37	Bulk	92	82	87	89	10	
14 May 2022 09:38:37	Bulk	90	87	88	86	6	Partial shadow on SP2 based on Figure 16.
14 May 2022 09:43:37	Bulk	90	87	87	85	5	
14 May 2022 09:48:37	Bulk	91	84	87	84	7	
14 May 2022 09:53:38	Bulk	88	88	89	83	5	
14 May 2022 09:58:37	Bulk	91	88	88	85	6	
14 May 2022 10:03:37	Bulk	94	92	89	80	14	
14 May 2022 10:08:37	Bulk	90	90	88	81	9	
14 May 2022 10:13:38	Bulk	90	91	88	80	11	
14 May 2022 10:18:37	Bulk	92	86	88	83	9	
14 May 2022 10:23:37	Bulk	89	94	90	92	5	
14 May 2022 10:28:37	Bulk	88	88	90	88	8	
14 May 2022 10:33:37	Bulk	92	88	87	88	4	
14 May 2022 10:38:37	Bulk	91	88	87	87	4	
14 May 2022 10:43:38	Bulk	89	95	80	88	15	
14 May 2022 10:48:37	Bulk	86	94	86	86	8	
14 May 2022 10:53:38	Bulk	88	88	89	87	1	
14 May 2022 10:58:37	Bulk	88	90	90	88	2	
14 May 2022 11:03:38	Bulk	88	91	88	88	3	
14 May 2022 11:08:37	Bulk	88	90	88	88	2	
14 May 2022 11:13:38	Bulk	94	81	95	81	14	
14 May 2022 11:18:37	Bulk	88	91	85	88	6	
14 May 2022 11:23:38	Bulk	96	84	84	84	12	No shadow based on Figure 16.
14 May 2022 11:28:37	Bulk	89	94	87	83	11	
14 May 2022 11:33:38	Bulk	89	93	92	89	4	
14 May 2022 11:38:37	Bulk	91	90	94	83	11	
14 May 2022 11:43:38	Bulk	88	88	91	85	6	
14 May 2022 11:48:37	Bulk	81	82	81	80	2	
14 May 2022 11:53:38	Bulk	91	89	88	89	3	
14 May 2022 11:58:37	Bulk	89	91	92	82	11	
14 May 2022 12:03:37	Bulk	89	93	90	82	11	
14 May 2022 12:08:37	Bulk	89	93	90	83	10	
14 May 2022 12:13:38	Bulk	81	84	91	81	10	
14 May 2022 12:18:37	Bulk	90	91	92	88	4	
14 May 2022 12:23:38	Bulk	57	49	57	57	8	
14 May 2022 12:28:37	Bulk	89	89	90	87	3	
14 May 2022 12:33:37	Bulk	95	85	92	85	10	
14 May 2022 12:38:37	Bulk	72	72	73	73	1	
14 May 2022 12:43:37	Bulk	91	91	92	88	4	
14 May 2022 12:48:37	Bulk	77	77	78	78	1	
14 May 2022 12:53:37	Bulk	84	84	86	86	2	
14 May 2022 12:58:37	Bulk	87	89	88	89	2	
14 May 2022 13:03:37	Bulk	88	85	99	85	14	
14 May 2022 13:08:37	Bulk	89	91	89	91	2	
14 May 2022 13:13:37	Bulk	89	91	89	87	4	
14 May 2022 13:18:37	Bulk	82	82	83	83	1	
14 May 2022 13:23:38	Bulk	57	57	58	58	1	
14 May 2022 13:28:37	Bulk	70	70	72	72	2	
14 May 2022 13:33:37	Bulk	90	89	90	90	1	Partial shadow on SP3 based on Figure 16.
14 May 2022 13:38:37	Bulk	92	89	89	85	7	
14 May 2022 13:43:37	Bulk	92	89	89	86	6	
14 May 2022 13:48:37	Bulk	82	93	91	91	11	
14 May 2022 13:53:37	Bulk	89	90	91	90	2	
14 May 2022 13:58:37	Bulk	93	90	91	90	3	
14 May 2022 14:03:37	Bulk	96	76	94	95	1	
14 May 2022 14:08:37	Bulk	89	79	92	91	13	
14 May 2022 14:13:37	Bulk	76	74	77	77	3	
14 May 2022 14:18:37	Bulk	88	91	96	93	8	
14 May 2022 14:23:37	Bulk	97	71	99	90	28	
14 May 2022 14:28:38	Bulk	92	82	98	87	16	
14 May 2022 14:33:37	Bulk	101	38	108	93	70	Partial shadow on SP3 and SP4 based on Figure 16.
14 May 2022 14:38:38	Bulk	87	86	93	93	7	
14 May 2022 14:43:37	Absorption	93	75	102	98	27	
14 May 2022 14:48:38	Absorption	86	87	95	94	8	
14 May 2022 14:53:37	Absorption	74	74	80	80	6	
14 May 2022 14:58:37	Absorption	89	40	100	97	60	
14 May 2022 15:03:37	Absorption	86	49	96	95	55	

**Table A3:** SP quantitative power values and deviation registered the 14 May 2022 from 15:08:37 to 15:08:37 hours.

Time Stamp UTC(+00:00)	Charge State	SP4 Power	SP3 Power	SP2 Power	SP1 Power	P_SPimax_t Deviation	Partial Shadow
14 May 2022 15:08:37	Absorption	83	55	89	92	37	Partial shadow on SP3 and SP4 based on Figure 16.
14 May 2022 15:13:38	Absorption	83	79	91	90	12	
14 May 2022 15:18:37	Absorption	81	80	89	89	9	
14 May 2022 15:23:37	Absorption	75	69	88	86	19	
14 May 2022 15:28:37	Absorption	78	64	86	85	22	
14 May 2022 15:33:37	Absorption	77	59	85	85	26	
14 May 2022 15:38:37	Absorption	74	61	83	83	22	
14 May 2022 15:43:37	Absorption	73	61	81	81	20	
14 May 2022 15:48:37	Absorption	70	62	78	47	16	
14 May 2022 15:53:37	Absorption	67	65	76	76	11	
14 May 2022 15:58:37	Absorption	65	63	74	74	11	
14 May 2022 16:03:37	Absorption	62	61	71	72	11	
14 May 2022 16:08:37	Absorption	60	58	69	69	11	
14 May 2022 16:13:38	Absorption	58	56	66	67	11	
14 May 2022 16:18:37	Absorption	55	54	64	64	10	
14 May 2022 16:23:38	Absorption	53	51	62	62	11	
14 May 2022 16:28:37	Absorption	46	49	60	60	11	
14 May 2022 16:33:38	Absorption	48	47	58	58	11	
14 May 2022 16:38:37	Absorption	44	43	54	54	11	
14 May 2022 16:43:38	Absorption	44	43	53	53	10	
14 May 2022 16:48:37	Absorption	41	39	50	50	11	
14 May 2022 16:53:38	Absorption	39	38	48	48	10	
14 May 2022 16:58:37	Absorption	36	35	44	45	10	
14 May 2022 17:03:37	Absorption	33	33	42	42	9	
14 May 2022 17:08:37	Absorption	31	31	39	40	9	
14 May 2022 17:13:38	Absorption	29	28	37	38	10	
